# Genome-wide Identification and Evolutionary Analysis of NBS-LRR Genes From *Secale cereale*

**DOI:** 10.3389/fgene.2021.771814

**Published:** 2021-11-09

**Authors:** Lan-Hua Qian, Yue Wang, Min Chen, Jia Liu, Rui-Sen Lu, Xin Zou, Xiao-Qin Sun, Yan-Mei Zhang

**Affiliations:** ^1^ Suzhou Polytechnic Institute of Agriculture, Suzhou, China; ^2^ Institute of Botany, Jiangsu Province and Chinese Academy of Sciences, Nanjing, China; ^3^ Seed Administrative Station of Suzhou, Suzhou, China

**Keywords:** *Secale cereale*, NBS-LRR gene, disease resistance, triticeae crops, evolutionary analysis

## Abstract

*Secale cereale* is an important crop in the Triticeae tribe of the Poaceae family, and it has unique agronomic characteristics and genome properties. It possesses resistance to many diseases and serves as an important resource for the breeding of other Triticeae crops. We performed a genome-wide study on *S. cereale* to identify the largest group of plant disease resistance genes (*R* genes), the nucleotide-binding site-leucine-rich repeat receptor (NBS-LRR) genes. In its genome, 582 NBS-LRR genes were identified, including one from the RNL subclass and 581 from the CNL subclass. The NBS-LRR gene number in the *S. cereale* genome is greater than that in barley and the diploid wheat genomes. *S. cereale* chromosome 4 contains the largest number of NBS-LRR genes among the seven chromosomes, which is different from the pattern in barley and the genomes B and D of wheat but similar to that in the genome A of wheat. Further synteny analysis suggests that more NBS-LRR genes on chromosome 4 have been inherited from a common ancestor by *S. cereale* and the wheat genome A than the wheat genomes B and D. Phylogenetic analysis revealed that at least 740 NBS-LRR lineages are present in the common ancestor of *S. cereale*, *Hordeum vulgare* and *Triticum urartu*. However, most of them have only been inherited by one or two species, with only 65 of them preserved in all three species. The *S. cereale* genome inherited 382 of these ancestral NBS-LRR lineages, but 120 of them have been lost in both *H. vulgare* and *T. urartu*. This study provides the full NBS-LRR profile of the *S. cereale* genome, which is a resource for *S. cereale* breeding and indicates that *S. cereale* can be an important material for the molecular breeding of other Triticeae crops.

## Introduction

Plants are affected by many biotic and abiotic stresses during their lifespan. In responding to these stressors, plants have evolved a two-layer immune system against pathogen infection (Jones and Dangl, 2006). The first layer of immunity recognizes conserved pathogen associated molecular patterns (PAMPs) through cell-surface located receptor-like proteins and receptor-like kinases and is termed PAMP-triggered immunity (PTI) (Jones and Dangl, 2006). To overcome the PTI process, some successful pathogens can release effector proteins into the intracellular part of plant cells and disturb the PTI process (Jones and Dangl, 2006). These effector proteins can be recognized directly or indirectly by intracellular proteins encoded by plant disease resistance genes (*R* genes), which will activate the second layer of plant immunity termed effector-triggered immunity (ETI) (Jones and Dangl, 2006). Among over 300 cloned *R* genes, more than 60% of them belong to the nucleotide-binding site leucine-rich repeat (NBS-LRR) gene family (Kourelis and van der Hoorn, 2018). The proteins encoded by typical NBS-LRR genes have two common domains, which are the central NBS domain and the C-terminal LRR domain (DeYoung and Innes, 2006). The sequences of the NBS domain encoded by different NBS-LRR genes are highly conserved (DeYoung and Innes, 2006). In contrast, the LRR domain of NBS-LRR proteins, which generally takes the role of pathogen recognition, is highly variable among different NBS-LRR proteins (DeYoung and Innes, 2006).

In angiosperms, NBS-LRR genes are divided into three subclasses based on their phylogenetic relationship (Shao et al., 2016). Characteristic N-terminal domains have been found for three NBS-LRR subclasses, including the toll-like/interference receptor/Resistance (TIR) domain, the coiled-coil (CC) domain and the RPW8 domain. Accordingly, the three NBS-LRR subclasses encoding these domains are named as TIR-NBS-LRR (TNL), CC-NBS-LRR (CNL) and RPW8-NBS-LRR (RNL) genes (Shao et al., 2016), respectively. The origin of NBS-LRR genes and the divergence of the three NBS-LRR subclasses can be traced to the common ancestor of the green lineage (Shao et al., 2019). After plant colonization of the land, NBS-LRR genes expanded greatly in land plant genomes (Liu et al., 2021), suggesting that NBS-LRR genes may have served as a major receptor of the plant immune system for millions of years. Importantly, nearly all NBS-LRR genes with a known function are involved in plant immunity (Kourelis and van der Hoorn, 2018).

Due to the specialized function of the NBS-LRR gene family in plant immunity and its high abundance in plant genomes, many studies have involved genome-wide identification of NBS-LRR genes from crops and their closely related non-crop species since early studies on the genomes of rice and *Arabidopsis thaliana* (Bai et al., 2002; Meyers et al., 2003). Hundreds of NBS-LRR genes have been identified from the genomes of rice, maize, wheat, barley and soybean (Liu et al., 2021). These studies provide fundamental resources for mining and utilization of functional NBS-LRR genes in crops. For example, by comparative analysis of NBS-LRR genes among different rice varieties and species from the Poaceae family, more than 100 NBS-LRR genes against rice blast have been cloned (Yang et al., 2013; Zhang et al., 2015; Guo et al., 2016).

*Secale cereale* together with common wheat (*Triticum aestivum*) and barley (*Hordeum vulgare*) are important crops in the Triticeae tribe of the Poaceae family. *S. cereale* has unique characteristics in both agronomic performance and genome properties (Crespo-Herrera et al., 2017). It has resistance to many fungal diseases that cause severe economic losses in wheat and other Triticeae crops (Singh et al., 2016). The 1RS chromosome arm of *S. cereale* has been transferred to the wheat genome, which has contributed to the control of powdery mildew and stripe rust diseases in worldwide wheat production (Szakacs et al., 2020). However, the exact genes that contribute to the resistance are unknown. Recent studies have identified NBS-LRR genes from barley and several wheat genomes, taking advantage of the published genomes (Andersen et al., 2020; Li Q. et al., 2021). However, the NBS-LRR profile of *S. cereale* has not been determined. Herein, the genome-wide identification and evolutionary analysis of NBS-LRR genes were performed on a recently released *S. cereale* genome (Li G. et al., 2021). The uncovered NBS-LRR gene profile in this study may serve as a resource for further mining and application of functional *R* genes in *S. cereale* and other Triticeae species.

## Materials and Methods

### Data Used in This Study

Genome sequences and annotation files of *S. cereale*, *T. aestivum* and *H. vulgare* were downloaded from https://bigd.big.ac.cn/, https://ftp.ensemblgenomes.org/pub/plants/release-51/fasta/triticum_aestivum/dna/, and http://doi.org/10.5447/ipk/2021/3, respectively. The NBS-LRR genes of *T. aestivum*, *T. urartu* and *H. vulgare* were retrieved from previous studies (Li Q. et al., 2021; Liu et al., 2021).

### Identification of NBS-LRR Genes

NBS-LRR gene identification was performed on the annotated proteins of *S. cereale* as described previously (Zhang et al., 2020). Briefly, the Hidden Markov Model (HMM) profile of the NB-ARC domain (Pfam accession number: PF00931) was used as a query to perform the HMMsearch using the HMMER-3.0 package against the protein sequences of *S. cereale* with an E-value setting of 1.0. Then, the obtained amino acid sequences were used as queries to perform a round BLASTp search against the protein sequences of *S. cereale* with an E-value setting of 1.0. The obtained protein sequences of BLASTp search were scanned by HMMscan against the Pfam-A database to confirm the presence of the NB-ARC domain (E-value set to 0.0001). Genes without a conserved NBS domain were removed from the datasets. All of the non-redundant candidate sequences were compared with the NCBI Conserved Domains Database (CDD) to further verify the CC, TIR (Pfam accession number: PF01582), RPW8 (Pfam accession number: PF05659), LRR and other integrated domains.

MEME analysis (Bailey et al., 2009) was performed to discover conserved motifs in the NB-ARC domain of the identified NBS-LRR genes. The number of displayed motifs was set to 20 with all other parameters at default settings.

### Distribution of NBS-LRR Genes on Different Chromosomes

The distribution of NBS-LRR genes on the chromosomes of the *S. cereale* genome was determined by extracting and parsing genomic locations of the NBS-LRR genes from the GFF3 annotation file. A sliding window analysis was performed with a window size of 250 kb to identify the number of genes that appeared in a cluster on a chromosome as described by Ameline-Torregrosa et al. (2008). If two successive annotated NBS-LRR genes were located within 250 kb on a chromosome, they were considered as clustered.

### Phylogenetic Analysis

Phylogenetic analysis of NBS-LRR genes was performed as described by Zhang et al. (2020). Briefly, amino acid sequences of the conserved NB-ARC domain of the NBS-LRR genes from *S. cereale*, *T. urartu* and *H. vulgare* were aligned using ClustalW with default options and then manually corrected in MEGA 7.0 (Kumar et al., 2016). Too short or extremely divergent sequences were excluded from the analysis to generate the finally matrix (Supplementary Dataset 1). Phylogenetic analysis was carried out with IQ-TREE using the maximum likelihood method after selecting the best-fit model using ModelFinder (Nguyen et al., 2015; Kalyaanamoorthy et al., 2017). Branch support values were estimated using UFBoot2 (Minh et al., 2013) and SH-aLRT tests (Anisimova and Gascuel, 2006). Reconciling the NBS-LRR phylogeny with the species tree was performed as described in a previous study (Shao et al., 2014) by using Notung software (Stolzer et al., 2012).

### Synteny and Gene Duplication Analysis

For cross-species synteny analysis, pair-wise inter-species all-against-all BLAST was performed for the *S. cereale*, *T. aestivum* and *H. vulgare* protein sequences. The obtained results and the GFF annotation file were then subjected to MCScanX (Wang et al., 2012) for inter-species synteny detection. For intra-species synteny analysis of *S. cereale*, pair-wise intra-species all-against-all BLAST was performed for the *S. cereale* protein sequences. The obtained results and the GFF annotation file were then subjected to MCScanX for intra-species microsynteny detection and determination of the gene duplication type (Wang et al., 2012). Microsynteny relationships were displayed using TBtools (Chen et al., 2020).

## Results

### Identification of NBS-LRR Genes in the *S. cereale* Genome

We identified 582 NBS-LRR genes (Supplementary Table S1) in the *S. cereale* genome accounting for more than 0.6% of the 86,991 annotated protein coding genes. The number of NBS-LRR genes in *S. cereale* is greater than that in the *H. vulgare* genome (467 genes) and slightly more than that in the diploid wheat *T. urartu* genome (537 genes) (Li Q. et al., 2021; Liu et al., 2021). This suggests that it is an important resource for Triticeae R gene mining. To assign these NBS-LRR genes to different subclasses, their amino acid sequences were subjected to BLASTp analysis against the well-annotated *A. thaliana* NBS-LRR proteins, as described previously (Zhang et al., 2016; Zhang et al., 2020). The results showed that 581 of the 582 *S. cereale* NBS-LRR genes belong to the CNL subclass, whereas only one gene was classified to the ADR1-lineage of the RNL subclass. This is consistent with the studies on *H. vulgare* and *T. urartu*, which also identified only one RNL gene in each of the two genomes (Li Q. et al., 2021; Liu et al., 2021), mirroring the conserved function of RNL genes. No TNL gene was detected in the *S. cereale* genome as have been observed in *H. vulgare* and *T. urartu*, supporting the theory that TNL genes were lost in the common ancestor of monocots (Liu et al., 2021).

### Domain Composition and Arrangement of *S. cereale* NBS-LRR Proteins

**FIGURE 1:**
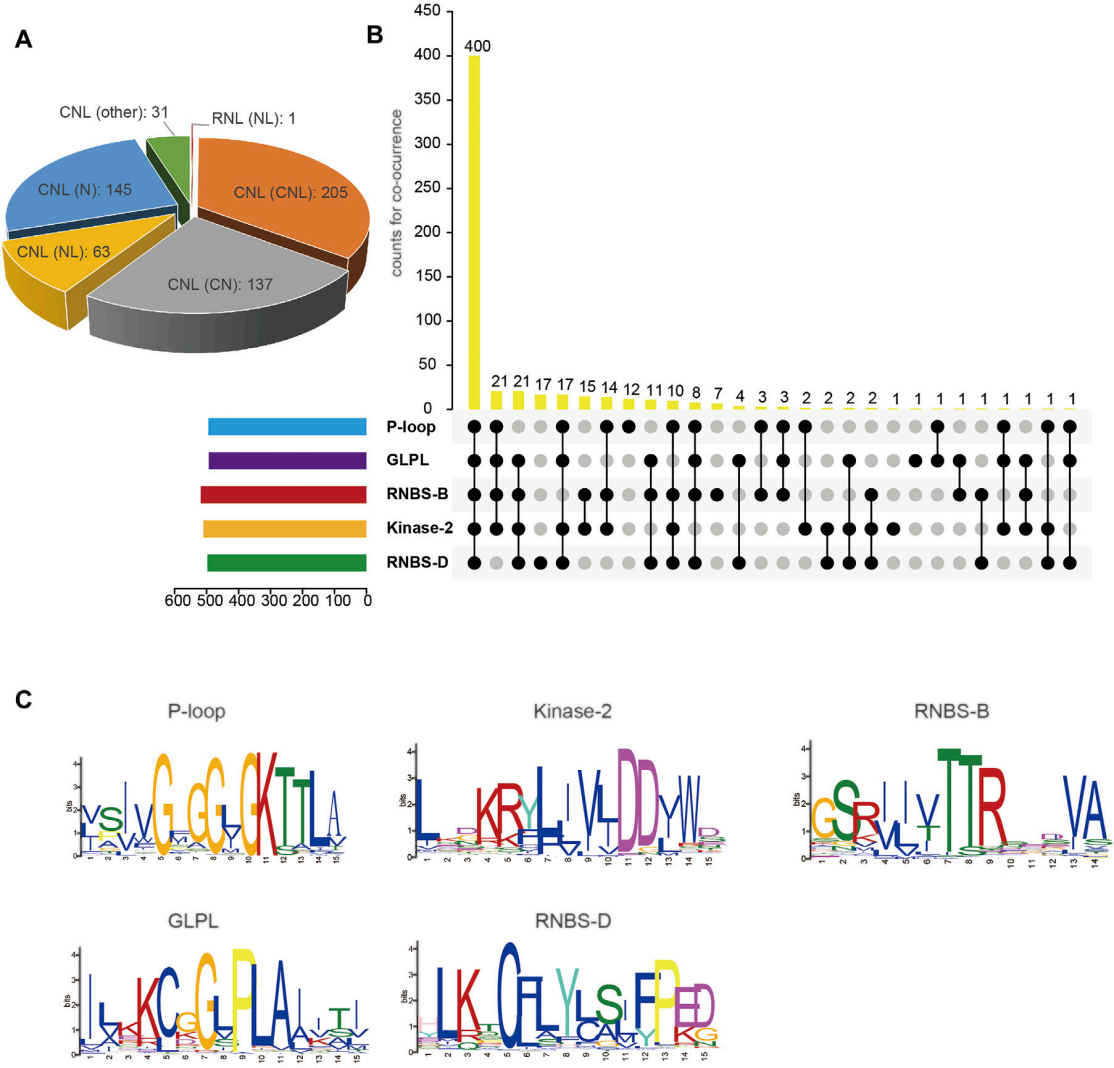
Classification and sequence features of NBS-LRR genes in *S. cereale*. **(A)** Classification and domain compositions of *S. cereale* NBS-LRR proteins. **(B)** Presence of five key motifs in the amino acid sequence of the NBS domain of *S. cereale* NBS-LRR protein. **(C)** Amino acid features of five key motifs in the amino acid sequence of the NBS domain of *S. cereale* NBS-LRR protein.

Analysis of the domain composition of the *S. cereale* NBS-LRR proteins revealed that not all of them have the characteristic domains at the N-terminal and the LRR domain at the C-terminal. In contrast, the NBS-LRR proteins show high domain composition and structure diversity (Figure 1 and Supplementary Table S1). The protein encoded by the single RNL gene contains both NBS and LRR domains but lacks a detectable RPW8 domain at the N-terminal. Among the 581 genes in the CNL subclass, 205 genes encode intact CNL proteins that simultaneously contain the typical N-terminal CC domain, the central NBS domain and the C-terminal LRR domain, accounting for 35% of all CNL genes (Figure 1A). There are 63 CNL genes encoding proteins without the N-terminal CC domain forming the NBS-LRR (NL) structure, and 137 CNL genes encoding proteins without the C-terminal LRR domain forming the NBS-LRR (NL) structure. We also found 145 genes encoding proteins that lack both N-terminal and C-terminal domains. The remaining 31 CNL genes have complicated domain structures (assigned to the “other” group in Figure 1A).

Additional integrated domains (IDs) were found at the N-terminal and/or the C-terminal of 49 of 581 CNL genes (Supplementary Table S1), forming the CNL-ID (23 genes), ID-CNL (25 genes) and ID-CNL-ID (one gene) structures. A total of 22 different IDs, including Jacalin, ZnF_BED and WRKY, were detected from 49 *S. cereale* NBS-LRR proteins, accounting for 8% of all NBS-LRR proteins.

### Detection of Key Motifs at the NBS Domain of *S. cereale* NBS-LRR Proteins

Several key motifs have been identified in the NBS domain of many NBS-LRR genes, including the P-loop, Kinase-2, RNBS-B, GLPL and RNBS-D (DeYoung and Innes, 2006). The distribution and sequence profile of these motifs in the NBS domain of *S. cereale* NBS-LRR proteins were detected by the Multiple Em for Motif Elicitation (MEME) suite (Bailey et al., 2009). The results showed that the NBS domain of 400 NBS-LRR proteins has all five motifs, accounting for 69% of all NBS-LRR proteins (Figure 1B). In contrast, the 182 remaining NBS-LRR proteins lack at least one of these motifs at the NBS domain, including 77 proteins that lost one motif, 37 proteins that lost two motifs, 29 that lost three motifs and 21 that lost four motifs. The ratio of NBS-LRR genes containing all five motifs at the NBS domain in *S. cereale* is higher than that in barley, for which only 283 NBS-LRR proteins preserve all five motifs at the NBS domain, accounting for 60% of all NBS-LRR proteins (Li Q. et al., 2021). Analyzing the sequence features of the motifs of CNL proteins (Figure 1C) revealed that the sequence profiles of Kinase-2 and RNBS-B show the characteristic feature of CNL genes “DDVW” and “TTR,” which was reported by Shao et al. (2016).

### Chromosomal Distribution of NBS-LRR Genes in the *S. cereale* Genome

**FIGURE 2:**
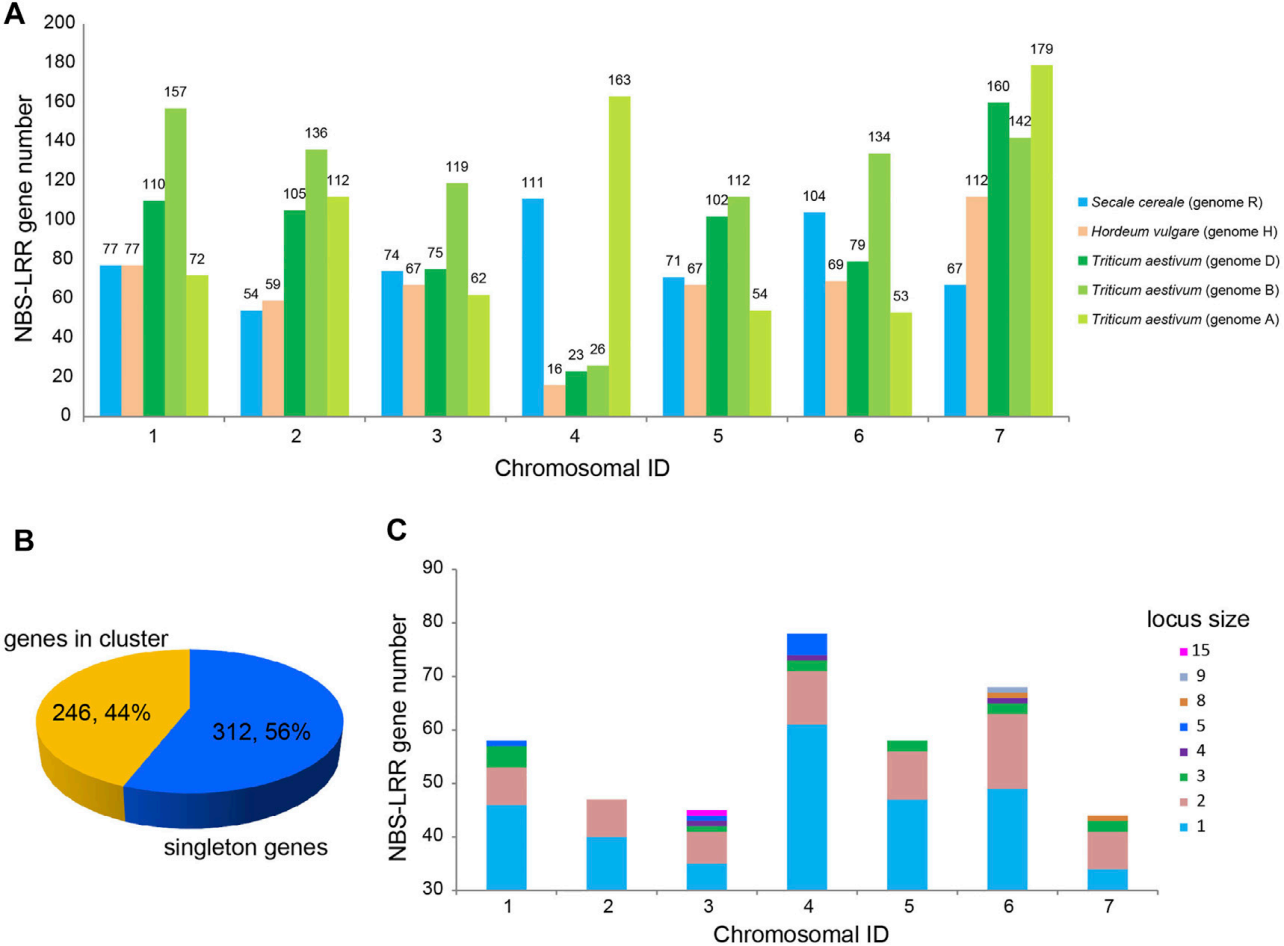
Chromosomal distribution of NBS-LRR genes in *S. cereale*. **(A)** Comparative analysis of chromosomal distribution of NBS-LRR genes in *S. cereale* ***,*** *H. vulgare* and *T. aestivum*. **(B)** Proportion of NBS-LRR genes appearing as singletons (blue) and clusters (yellow) in the *S. cereale* genome. **(C)** Proportion of NBS-LRR genes appearing as singletons or different-size clusters on each chromosome of *S. cereale.*

To determine the distribution of the 582 NBS-LRR genes on the seven chromosomes of *S. cereale*, the physical locations of NBS-LRR genes were retrieved from the GFF3 annotation file. The result showed that *S. cereale* NBS-LRR genes are unevenly distributed on the seven chromosomes (Figure 2). Among the 582 NBS-LRR genes identified from the *S. cereale* genome, 558 were anchored on the seven chromosomes in the annotation file, whereas the chromosomal location of 24 NBS-LRR genes was not determined by the genomic annotation. Chromosome 4 has the most NBS-LRR genes (111), and this is nearly two times the number of NBS-LRR genes on chromosome 2 (54). Chromosomes 1, 3, 5, 6 and 7 have 77, 74, 71, 104 and 61 NBS-LRR genes, respectively. We also compared the chromosome distribution pattern of NBS-LRR genes in the *S. cereale* genome with that in the barley and wheat genomes (Li Q. et al., 2021; Liu et al., 2021), because the three species have the same number of chromosomes and have only diverged from each other for about 10 myr. Since the diploid wheat *T. urartu* with AA genome does not have a chromosomal level gff annotation file, we used the data of the hexaploid wheat *T. aestivum*, which contain information of all three sets of chromosomes (AABBDD) of wheat (Liu et al., 2021). The results showed that chromosome 4, which has the largest number of NBS-LRR genes among all chromosomes in *S. cereale*, only has 16, 23 and 26 NBS-LRR genes in barley and the B and D genomes of wheat, representing the chromosome with the fewest NBS-LRR genes in these genomes. In contrast, we found that chromosome 4 of the wheat A genome has 163 NBS-LRR genes, ranking second among the seven chromosomes in the A genome. This feature is quite similar to that found in *S. cereale*.

NBS-LRR genes on chromosomes can appear as a singleton locus or a cluster locus. The NBS-LRR cluster locus is defined as several NBS-LRR genes with an interval of less than 250 kb (Ameline-Torregrosa et al., 2008). Based on the physical locations, the 558 NBS-LRR genes on the seven *S. cereale* chromosomes were classified into 398 loci, including 312 singletons and 86 clusters (Figure 2). This suggested that 246 (44%) of the NBS-LRR genes are present in the 86 clusters, with an average of three genes per cluster. Among the 86 loci clusters, the smallest ones only have two NBS-LRR genes, including 7, 7, 6, 9, 9, 14 and 7 such loci on chromosome 1–7 (Supplementary Table S1). The largest cluster is located on chromosome 3, which contains 15 NBS-LRR genes.

### Gene Duplication Type of *Secale cereale* NBS-LRR Genes

**FIGURE 3:**
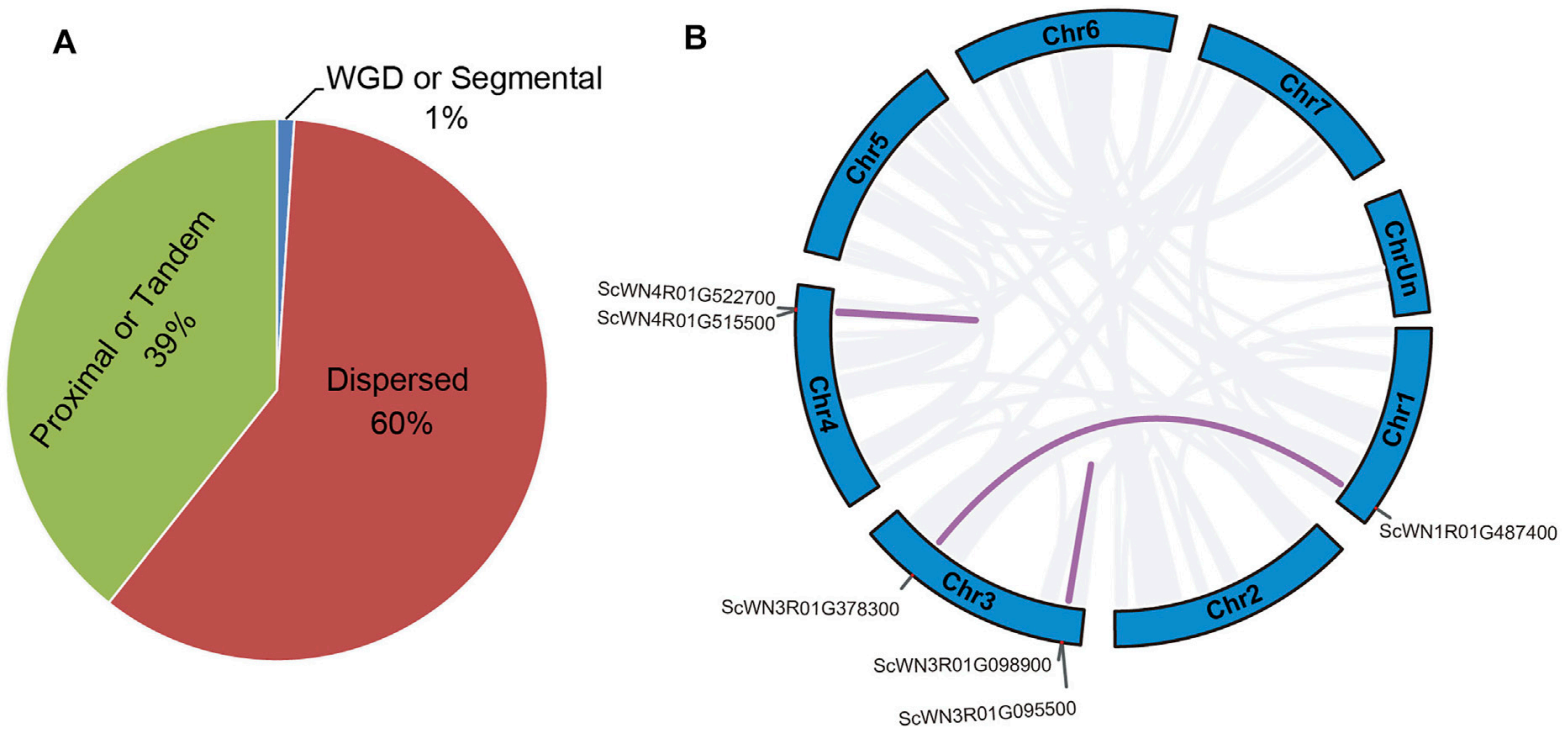
Duplication type for NBS-LRR genes in *S. cereale*. **(A)** Proportion of NBS-LRR genes with different duplication types. **(B)** Segmental duplicated NBS-LRR gene pairs in *S. cereale.*

Different types of gene duplication may contribute to the NBS-LRR gene family expansion. Our analysis revealed that about 39% of NBS-LRR genes in *S. cereale* were duplicated through tandem or proximal duplications; over 60% of NBS-LRR genes resulted from dispersed duplication, and only 1% (six genes) were generated from segmental duplications (Figure 3A). The six segmental duplicated genes form three gene pairs (Figure 3B). One pair involving SCWN1R01G487400 and SCWN3R01G378300 occurred between chromosomes 1 and 3, whereas the remaining two events were intra-chromosomal duplications on chromosomes 3 (SCWN3R01G095500 and SCWN3R01G098900) and 4 (SCWN4R01G515500 and SCWN4R01G522700), respectively.

### Interspecies Synteny of NBS-LRR Genes Among Three Triticeae Crops

**FIGURE 4:**
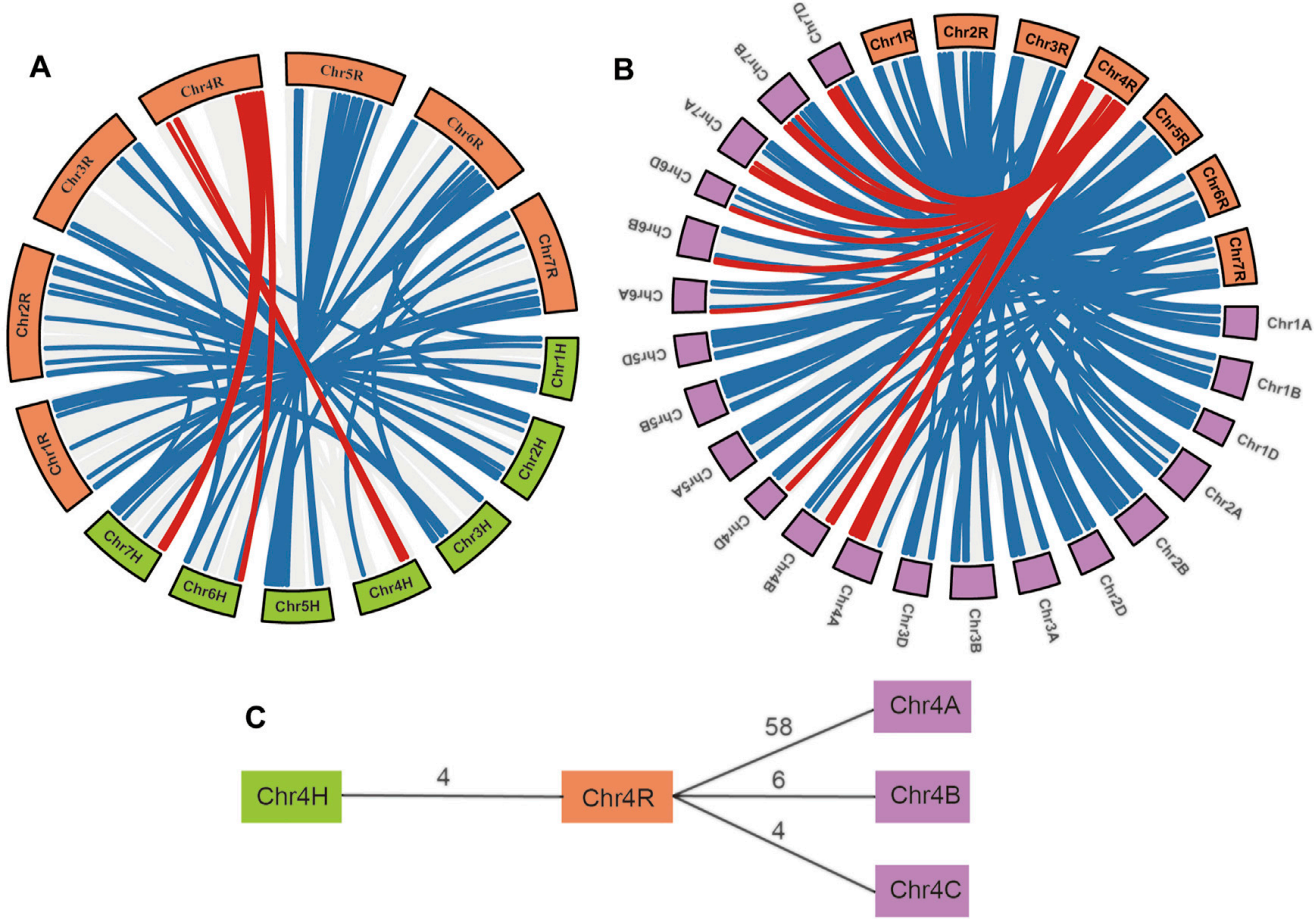
Cross-species synteny of *S. cereale* NBS-LRR genes. **(A)** Cross-species synteny between *S. cereale* and *H. vulgare*. **(B)**. Cross-species synteny between *S. cereale* and *T. aestivum*. Gene pairs involving NBS-LRR genes on chromosome 4 of *S. cereale* were labeled with a red line. **(C)** Comparative analysis of synteny NBS-LRR pairs between *S. cereale* and *H. vulgare* with synteny NBS-LRR pairs between *S. cereale* and *T. aestivum*.

Gene pairs within syntenic chromosome blocks are highly confidential orthologous genes among species. Synteny analysis of *S. cereale* with the barley and wheat genomes revealed that 143 NBS-LRR gene pairs between *S. cereale* and barley are located on syntenic chromosomal blocks of the two species (Figure 4A and Supplementary Table S2). Among the three wheat genomes, genome A has the most (245) syntenic NBS-LRR gene pairs with *S. cereale*, whereas genomes B and D have 216 and 209 syntenic NBS-LRR gene pairs, respectively (Figure 4B and Supplementary Table S2). Notably, consistent with the larger number of NBS-LRR genes on chromosome 4A than on chromosomes 4B and 4D, chromosome 4A also has 58 syntenic NBS-LRR gene pairs with chromosome 4R in *S. cereale*, which is much larger than the six gene pairs and four gene pairs between 4R with 4B and 4R with 4D, respectively (Figure 4B and C). This indicates that the larger number of NBS-LRR genes on chromosomes 4R and 4A is not likely a consequence of independent NBS-LRR expansion in the two genomes, but a result of convergent retention of ancestral NBS-LRR gene loci that were present in the common ancestor of the three Triticeae crops. Also, potential gene introgression may have contributed to this profile.

### Phylogenetic Analysis of NBS-LRR Genes From the Three Triticeae Crops

**FIGURE 5:**
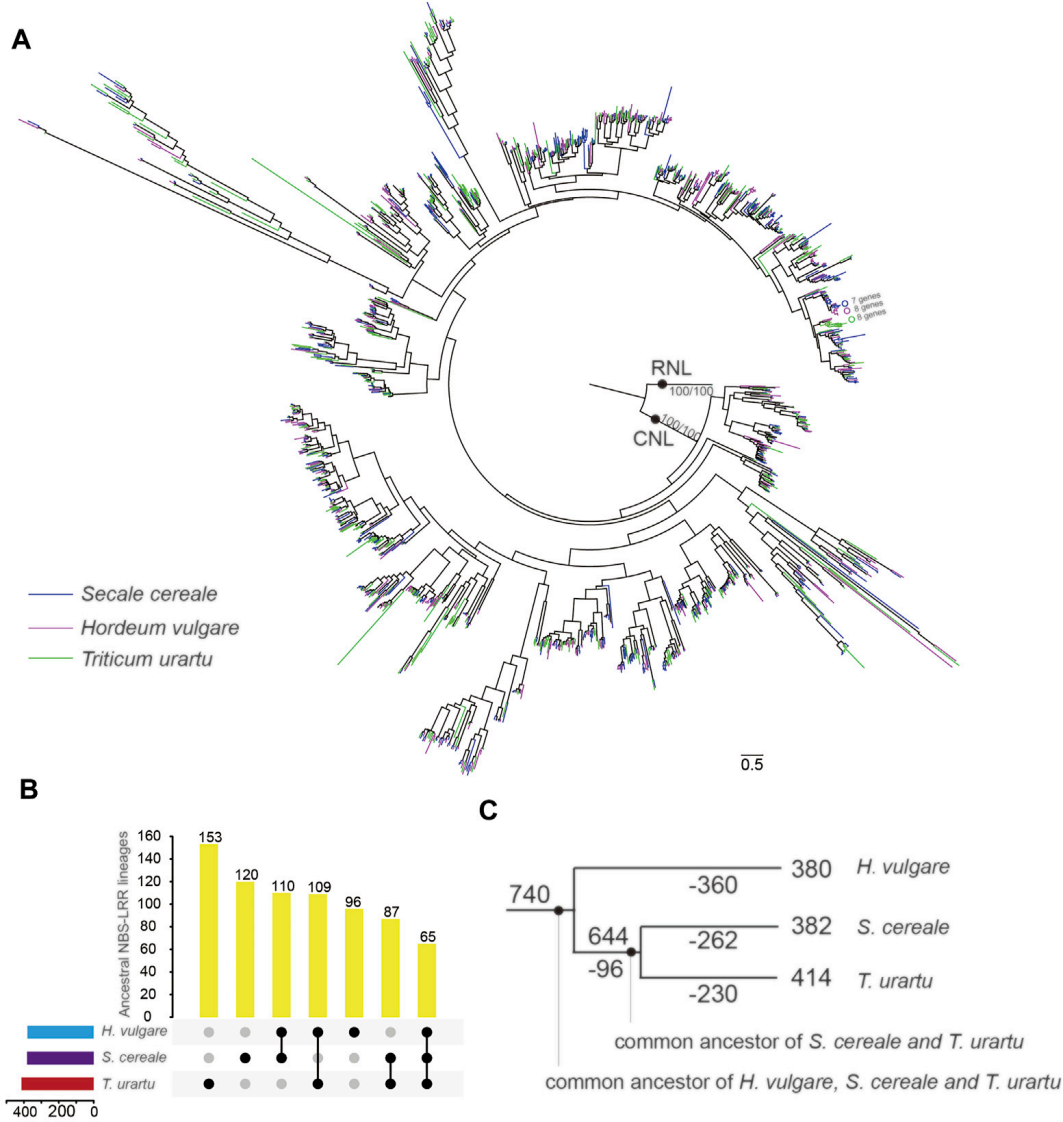
Phylogenetic and evolutionary analysis of NBS-LRR genes in *S. cereale*. **(A)** A phylogeny for NBS-LRR genes from *S. cereale* ***,*** *H. vulgare* and *T. urartu* constructed based on the conserved NBS domain. Branch support values obtained from SH-aLRT (%) and UFBoot2 (%) are labeled on basal nodes. **(B)** Distribution of ancestral NBS-LRR lineages among the three species. **(C)** The differential inheritance of 740 predicted ancestral NBS-LRR lineages during species speciation. Ancestral NBS-LRR lineage numbers on each node/branch are indicated. Numbers of lineage loss events are indicated by numbers with ‘-’ on each node/branch.

To trace the evolutionary history of *S. cereale* NBS-LRR genes, phylogenetic analysis was performed by incorporating NBS-LRR genes from *H. vulgare* and *T. urartu* (Li Q. et al., 2021; Liu et al., 2021). The phylogenetic result (Figure 5 and Supplementary Figure S1) showed that the three RNL genes from the three species form an independent clade that shares the same topology with the species tree with a high support value. This suggests a highly conserved evolutionary pattern of RNL genes. CNL genes from the three species form another highly supported clade. The topology supports that RNL and CNL subclasses diverged anciently in NBS-LRR gene evolution (Shao et al., 2019). The phylogeny shows the species-specific expansion of some CNL lineages when genes from the three species are labeled with different colors. As shown in Figure 5A, branches containing seven to eight NBS-LRR genes from a single species can be detected due to species-specific gene duplication.

Frequent gene losses and gains can be further resolved by resolving the gene tree with the phylogenetic relationship of the three species. The results showed that NBS-LRR genes from the three species can be traced to 740 ancestral lineages in their common ancestor approximately 15 myr ago (Figure S1). Among them, only 60 ancestral NBS-LRR lineages were inherited by all three species. Most of them conservatively evolved in each species without large duplication (Figure S1). In contrast, more than 90% (680) of ancestral NBS-LRR lineages were only inherited by one or two species after speciation (Figure 5B), including 380, 382 and 414 ancestral NBS-LRR lineages inherited by *H. vulgar*, *S. cereale* and *T. urartu*, respectively. Tracing the dynamics of the NBS-LRR gene evolutionary history revealed that 96 of the ancestral NBS-LRR lineages were lost in the common ancestor of *S. cereale* and *T. urartu* after it separated from *H. vulgare*, while 654 ancestral NBS-LRR lineages were preserved (Figure 5C). However, after the separation of *S. cereale* and *T. urartu*, a large proportion of ancestral NBS-LRR lineages experienced further gene loss, resulting in 492 ancestral NBS-LRR lineages being lost in either of the two species (262 in *S. cereale* and 230 *T. urartu*), while only 152 ancestral NBS-LRR lineages were shared by the two species (Figures 5B,C). The results indicate that NBS-LRR diversity among the three species is largely different due to their differential inheritance of ancestral lineages. Similar to the consistently occurring gene losses, gene duplication also frequently occurred during speciation, which contributed to the current NBS-LRR gene profile in the three species.

## Discussion

Since the first *R* gene Hm1 resistant to the fungus *Cochliobolus carbonum* race 1 was cloned from maize nearly 30 years ago, over 300 functional *R* genes have been identified. Most of these are from plant species that are important crops including rice, wheat and tomato (Johal and Briggs, 1992; Kourelis and van der Hoorn, 2018). Characterization of functional *R* genes was dependent on traditional map-based cloning methods in earlier studies. With the development of DNA sequencing technology, many crops have now been sequenced. The availability of these genomic resources has greatly accelerated the mining of functional *R* genes in economically important plants (Sanchez-Martin et al., 2016; Witek et al., 2016; Liu et al., 2020; Walkowiak et al., 2020) and has initiated a new era of molecular breeding (Poland and Rutkoski, 2016).

Most known *R* genes belong to the NBS-LRR gene family (Kourelis and van der Hoorn, 2018). The typical domain composition and high sequence similarity of genes in this gene family have enabled batch identification of NBS-LRR genes at the whole genome scale (Bai et al., 2002; Meyers et al., 2003). Large-scale screens of functional *R* genes from the NBS-LRR gene family conducted in the rice genome have enabled the identification of dozens of *R* genes that are active against the fungal pathogen *Magnaporthe oryzae* (Zhang et al., 2015; Guo et al., 2016), which is much more efficient than the traditional method. Genome-wide comparative analysis of NBS-LRR genes among several grass family species also helped in the identification of additional functional genes against *M. oryzae* in close-relatives of rice, including maize, sorghum and *Brachypodium*. This indicates that phylogenetically related species are important resources for *R* gene mining of crops (Yang et al., 2013).

The Triticeae is a tribe of the Poaceae family that contains many important grain crops, including wheat, barley, *S. cereale* and triticale. These crops are cultivated worldwide and are frequently challenged by pathogens and pests during their growth. Although resistance breeding has improved the performance of Triticeae crops (Forster, 1992; Delventhal et al., 2017), only a few functional *R* genes have been cloned from these crops (Kourelis and van der Hoorn, 2018). The recently released reference genome and pan-genome of wheat and barley have accelerated the identification of functional *R* genes more efficiently in these species (Jayakodi et al., 2020; Walkowiak et al., 2020). For example, a CNL gene resistant to the orange wheat blossom midge (OWBM, *Sitodiplosis mosellana* Géhin) has recently been cloned by analysis of the wheat pan-genome (Walkowiak et al., 2020). Four stem rust resistance genes have been cloned by combining association genetics with *R* gene enrichment sequencing in wild diploid wheat (Arora et al., 2019). Two CNL genes introduced from tall wheat grass (*Thinopyrum ponticum*), Sr26 and Sr61, against stem rust have been cloned from wheat by mutational genomics and targeted exome capture methods (Zhang et al., 2021). All of the above studies have benefited from understanding the NBS-LRR profile in the genomes.

Besides the wild wheat resources, both barley and *S. cereale* have been used to transfer genetic materials to wheat to improve its quality. The disease-resistance genes carried by the 1RS chromosome arm of *S. cereale* have been transferred to the wheat genome to confer resistance against powdery mildew and stripe rust diseases (Szakacs et al., 2020). However, without the genomic information, the *R* gene profile on the transferred chromosomal segment is unclear. In this study, genome-wide analysis identified 582 NBS-LRR genes in *S. cereale*. The number of NBS-LRR genes in *S. cereale* is larger than that in barley and *T. urartu* (Li G. et al., 2021). The high NBS-LRR gene number indicated that *S. cereale* would be an important resource for mining and transfer of functional *R* genes to barley and wheat. The distribution of NBS-LRR genes on different chromosomes of *S. cereale* was determined and serves as fundamental molecular information for the introduction of chromosomal segments from *S. cereale* to other Triticeae crops. An interesting finding is that both the chromosome 4 of *S. cereale* and the A genome of wheat retained a larger number of NBS-LRR genes than barley and genomes B and D of wheat. This result highlights chromosome 4 of *S. cereale* as an important resource for disease resistance breeding and also implies that chromosome 4 of wheat genome A has experienced a different evolutionary history compared to genomes B and D, at least for the NBS-LRR genes.

Although the number of NBS-LRR genes in the three Triticeae does not differ dramatically, a large proportion of the NBS-LRR genes from the three species are inherited from different ancestral NBS-LRR lineages that diverged in the common ancestor of the three species. The result suggested that the NBS-LRR diversity would be significantly increased if the three Triticeae species are considered as a ‘Triticeae NBS-LRR gene pool’ in resistance-gene mining. The high abundance of NBS-LRR genes in *S. cereale* and their distinct genetic origin with NBS-LRR genes in barley and wheat suggest that *S. cereale* could be an important resource for obtaining functional NBS-LRR genes for molecular breeding. This also provides a molecular basis for developing *S. cereale* as a donor material for Triticeae breeding.

In conclusion, we uncovered the NBS-LRR profile in *S. cereale* and compared the chromosomal distribution and evolutionary history of this gene family in three Triticeae species. This information provides a fundamental resource for mining functional *R* genes from *S. cereale*. Since cross-species transformation of genomic segments has been frequently used for the molecular breeding of Triticeae species, the NBS-LRR profile of *S. cereale* expands the gene pool for Triticeae molecular breeding.

## Data Availability

The original contributions presented in the study are included in the article/Supplementary Material, further inquiries can be directed to the corresponding authors.
