# Supplementary material for: Genome-wide Identification and Evolutionary Analysis of NBS-LRR Genes From Secale cereale
Source: Front Genet. 2021 Nov 9;12:771814. doi: 10.3389/fgene.2021.771814 (PMC8630680; doi:10.3389/fgene.2021.771814)
Supplement: Supplementary file 4 [file DataSheet1.PDF]

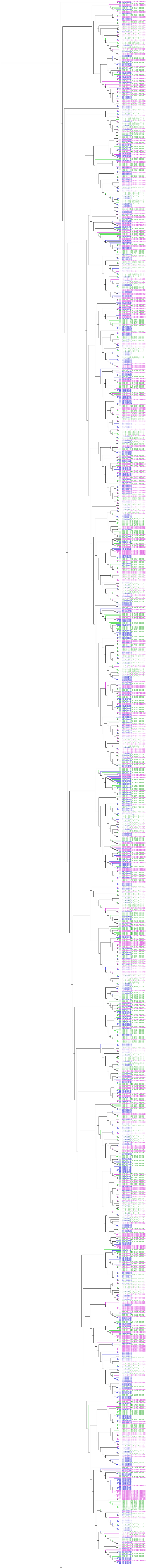
0.0
